# Functional and structural asymmetry in primary motor cortex in Asperger syndrome: a navigated TMS and imaging study

**DOI:** 10.1007/s10548-019-00704-0

**Published:** 2019-04-04

**Authors:** Laura Säisänen, Sara Määttä, Petro Julkunen, Eini Niskanen, Elisa Kallioniemi, Heidi Gröhn, Samuli Kemppainen, Timo A. Lakka, Niina Lintu, Aino-Maija Eloranta, Ritva Vanninen, Ismo Makkonen, Mervi Könönen

**Affiliations:** 1 Department of Clinical Neurophysiology, Institute of Clinical Medicine, Faculty of Health Sciences, University of Eastern Finland, Kuopio, Finland; 2 Department of Clinical Neurophysiology, Kuopio University Hospital, Kuopio, Finland; 3 Department of Applied Physics, University of Eastern Finland, Kuopio, Finland; 4 Department of Psychiatry, UT Southwestern Medical Center, Dallas, USA; 5 Department of Clinical Physiology and Nuclear Medicine, Kuopio University Hospital, Kuopio, Finland; 6 Institute of Biomedicine, School of Medicine, University of Eastern Finland, Kuopio Campus, Finland; 7 Kuopio Research Institute of Exercise Medicine, Kuopio, Finland; 8 Institute of Public Health and Clinical Nutrition, School of Medicine, University of Eastern Finland, Kuopio, Finland; 9 Department of Clinical Radiology, Kuopio University Hospital, Kuopio, Finland; 10 Department of Clinical Radiology, Institute of Clinical Medicine, Faculty of Health Sciences, University of Eastern Finland, Kuopio, Finland; 11 Department of Child Neurology, Kuopio University Hospital, Kuopio, Finland

**Keywords:** Navigated TMS, Asperger syndrome, Hemispheric asymmetry, Motor mapping, Neuroimaging, Voxel-based morphometry, Cortical plasticity, Brain reorganization

## Abstract

Motor functions are frequently impaired in Asperger syndrome (AS). In this study, we examined the motor cortex structure and function using navigated transcranial magnetic stimulation (nTMS) and voxel-based morphometry (VBM) and correlated the results with the box and block test (BBT) of manual dexterity and physical activity in eight boys with AS, aged 8–11 years, and their matched controls. With nTMS, we found less focused cortical representation areas of distinct hand muscles in AS. There was hemispheric asymmetry in the motor maps, silent period duration and active MEP latency in the AS group, but not in controls. Exploratory VBM analysis revealed less gray matter in the left postcentral gyrus, especially in the face area, and less white matter in the precentral area in AS as compared to controls. On the contrary, in the right leg area, subjects with AS displayed an increased density of gray matter. The structural findings of the left hemisphere correlated negatively with BBT score in controls, whereas the structure of the right hemisphere in the AS group correlated positively with motor function as assessed by BBT. These preliminary functional (neurophysiological and behavioral) findings are indicative of asymmetry, and co-existing structural alterations may reflect the motor impairments causing the deteriorations in manual dexterity and other motor functions commonly encountered in children with AS.

**Electronic supplementary material:**

The online version of this article (10.1007/s10548-019-00704-0) contains supplementary material, which is available to authorized users.

## Introduction

Asperger syndrome (AS) is a neurodevelopmental condition belonging to the autism spectrum disorders (ASD). The diagnostic criteria of ASD include persistent deficits in social communication and interaction, as well as restricted, repetitive patterns of behavior, interests, or activities. There is no delay in language development or any significant cognitive delay in AS.

However, motor dysfunction such as motor clumsiness, awkwardness and motor learning delays are often present in children with ASD appearing early in life (Enticott et al. 2013). Since motor dysfunctions are more easily quantifiable and better reproduced than the more complex communicative and social behaviors, their assessment may help in identifying and classifying children with the disorder (Behere et al. 2012). There are also minor deficits, so called neurological soft signs, that can be taken into consideration when assessing the motor functions in ASD (D’Agati et al. 2018). These, according to neurodevelopmental model cannot be linked to specific cerebral lesions. They are commonly observed in typically developing younger children, but their persistence into later childhood and adolescence is linked with an increased risk of psychiatric disorders (D’Agati et al. 2018). The motor problems encountered in AS have been postulated to be related to poor sensorimotor integration and to the higher level of motor planning, though the motor learning processes and the underlying “motor machinery” appear to function normally (Gowen and Hamilton 2013; Paquet et al. 2016). The motor, social and communication deficits may be linked traits that result from a dysfunction in parallel neural circuits (Papadopoulos et al. 2012; Barbeau et al. 2015). In addition, motor impairments may contribute to social impairments in ASD (Bhat et al. 2011). Therefore, it is essential to clarify the neural mechanisms underlying motor development and its dysfunction in ASD as they are the basis for the neurobiological background of the disorder.

Cortical motor function can be studied non-invasively using transcranial magnetic stimulation (TMS). Motor responses can be measured from the peripheral muscles; this allows a direct evaluation of cortical excitability and inhibition. This data cannot be obtained with any other imaging method. TMS has been shown to be applicable in pediatric population in pioneer studies (Lin and Pascual-Leone 2002; Nezu et al. 1997; Müller et al. 1997; Garvey et al. 2003) and later on (Frye et al. 2008; Kaye and Rotenberg 2017; Säisänen et al. 2018). In ASD, the motor threshold (MT), a measure of corticospinal excitability, appears to be unaffected (Oberman et al. 2015). Furthermore, there do not seem to be any alterations in motor-evoked potential (MEP) amplitudes in ASD (Enticott et al. 2010, 2013). Paired-pulse TMS with short-interval cortical inhibition (SICI) protocols can probe the inhibitory, GABAergic mechanisms, that have been postulated to be involved in the neurophysiology of ASD. There do not appear to be any clear deficits in GABA-A activity (Jung et al. 2013), but a reduced SICI may be present in some subgroups in the heterogeneous population of ASD, such as in the left hemisphere in those individuals with an early language delay (Enticott et al. 2010, 2013). The studies examining inhibitory GABA-B activity by silent period measurements have also shown controversial results. A trend towards reduced cortical silent period duration was shown in individuals with ASD (Theoret et al. 2005), but this was not confirmed in a later study (Enticott et al. 2013).

Navigated TMS (nTMS) incorporates magnetic resonance imaging (MRI) with TMS and provides continuous visualization of the anatomical site being stimulated (Ruohonen and Karhu 2010). The functional motor mapping delineates discrete representation areas of individual muscles. For example, it can be used to locate possibly reorganized motor areas in presurgical evaluation (Säisänen et al. 2010; Vitikainen et al. 2013; Krieg et al. 2014), stroke (Nudo et al. 1996), chronic pain (Schabrun et al. 2014), or to examine the neuroplasticity in healthy individuals (Vaalto et al. 2013). Multichannel electromyography (EMG) can be used to specify the representation areas of distal and proximal muscles and their relationships, for example, aberrant cortical organization or an unusual overlap (Marconi et al. 2007). In addition to the primary motor cortex (M1), also secondary motor areas involved in movement initiation and sensory guidance of movement (Shafer et al. 2017) can be mapped (Teitti et al. 2008). One of the notable benefits of nTMS is its excellent spatial resolution that ensures that the intended anatomical area is stimulated and that the responses are not merely induced because of electric field spreading. The method also optimizes the stimulation coil tilt and direction of the induced electric field to fit the anatomy and brain structure of each participant (Julkunen et al. 2009). Therefore, nTMS is able to provide new insights into the disease mechanisms responsible for the sometimes subtle and varying types of motor problems in ASD (Paquet et al. 2016). nTMS has not been previously applied in ASD subjects.

The structural MRI findings related to motor functions in ASD have been contradictory. A meta-analysis of voxel-based morphometry (VBM) studies focusing on pericentral regions has reported a decrease in gray matter (GM) in the left precentral gyrus (Cauda et al. 2011; Nickl-Jockschat et al. 2012) or left postcentral gyrus (DeRamus and Kana 2015). Increases in the GM have been found in the right or bilateral pre- and postcentral gyri (Rojas et al. 2006; DeRamus and Kana 2015; Mahajan et al. 2016). Evidence from a diffusion tensor imaging (DTI) study pointed towards impaired connectivity between the motor and somatosensory homunculus in adults with ASD (Thompson et al. 2017). These alterations were associated with poor manual dexterity. They also found evidence of interhemispheric differences.

The relationship between neuroanatomy and neurophysiology is a fundamental issue in neuroscience and of clinical relevance (Conde et al. 2012; Dayan et al. 2018; Kearney-Ramos et al. 2018). In this study we used a combination of TMS and structural MRI. Multimodal approach with information from different neuroimaging modalities provides a more comprehensive picture of the brain (Siebner et al. 2009).

In this study, our goal was to first address whether there are functional alterations in the motor cortex (representation areas or overlap of distal and proximal muscles) in AS in early adolescence that could be detected using a neurophysiological method of nTMS mapping, and considered as alterations in neuroplasticity. Secondly, we correlated these changes to the behaviorally assessed motor impairment. Finally, we integrated these functional alterations with exploratory neuroanatomical imaging. Both hemispheres were examined to specifically look at the asymmetry, since atypical rightward lateralization of motor circuit functional connectivity associated with motor deficits has also been observed with resting state functional MRI (Floris et al. 2016).

## Materials and methods

The study was approved by the Research Ethics Committee of the Hospital District of Northern Savo (48/2010) and was conducted in Kuopio University Hospital between 2010 and 2012. All participants and their parents provided informed consent.

### Participants and study design

The inclusion criteria were diagnosis of AS (ICD-10 diagnosis number F84.5) and year of birth between 2000 and 2002. In the catchment area of Northern Savo, the search from the Kuopio University Hospital patient register identified 24 eligible boys but only one girl. Thus we decided to recruit only boys. Children with concomitant neurologic or psychiatric diagnosis or medication affecting the central nervous system were excluded. Ultimately, nine boys (age range from 8 years and 4 months to 11 years and 7 months) were recruited but one of these boys had to be excluded since he did not meet the diagnostic criteria. The diagnosis of ASD was assessed by a multi-professional child psychiatry working group according to prevailing guidelines ICD-10 and DSM-IV and using neuropsychological test batteries and screening tools. The medical records of the candidates for the study were evaluated by a child neurologist, and AS was determined by ensuring the normal development of language abilities during the first 3 years of life. During the study, the parents were asked to fill in the Autism Spectrum Quotient (Auyeung et al. 2008) and Ehlers-Gillberg (Ehlers et al. 1999) screening questionnaires which were further checked together with a child neurologist during an interview. Eight boys matched individually for age, height and weight were selected and recruited as control subjects from a population sample of children participating in the Physical Activity and Nutrition in Children (PANIC) Study (Eloranta et al. 2012) being conducted in the Institute of Biomedicine, University of Eastern Finland. The exclusion criteria were common contraindications to MRI and TMS (Rossi et al. 2009).

### Magnetic resonance imaging (MRI)

Subjects were scanned in a 3.0 T scanner (Philips Achieva X, Philips Healthcare, Eindhoven, The Netherlands). Structural three-dimensional T1-weighted MR-images were acquired (TR 8.07 ms, TE 3.7 ms, flip angle 8°, 1 × 1 × 1 mm^3^ resolution) for nTMS and for the VBM analysis. The imaging took approximately half an hour in total. An experienced neuroradiologist evaluated the MR images for any abnormalities.

### Navigated transcranial magnetic stimulation (nTMS)

TMS was performed with an eXimia stimulator and a biphasic figure-of-eight coil combined with a navigation system (3.2.2 research version, Nexstim Plc., Helsinki, Finland). TMS-induced MEPs were recorded from peripheral muscles using disposable Ag-AgCl surface electrodes placed on abductor pollicis brevis (APB), abductor digiti minimi (ADM), first dorsal interosseus (FDI), extensor carpi radialis (ECR), flexor carpi radialis (FCR), and biceps brachii (BB) using a belly-tendon montage. Throughout the measurement, muscle activity was monitored on-line and recorded by stimulus-locked EMG (Nexstim Plc., Helsinki, Finland). The TMS session took approximately 2 h in total.

During the experiment, both hemispheres were stimulated in a randomized order. First, the optimal cortical representation site of the APB was determined (Säisänen et al. 2008a, b). At that site, using the aiming tool, the individual resting MT (rMT) for APB was determined using a threshold hunting paradigm (TMS Motor Threshold Assessment Tool 2.0) (Awiszus 2003; Awiszus and Borckardt 2012). Eleven MEPs were recorded from the APB using a stimulus intensity of 120% of the rMT both at rest and during slight muscle activation. Seven silent periods were recorded according to the previously described protocol at 120% of the rMT (Säisänen et al. 2008a, b). The mapping of motor representation areas of all recorded muscles was performed at a stimulation intensity of 110% of the rMT for APB (Kallioniemi and Julkunen 2016). A grid with line spacing of 5 mm was visualized on the surface of the brain for guidance during the mapping procedure. One stimulus was administered per square, and the stimulated area was extended until the borders of the representation area were reached where no more MEPs were elicited. The induced electric current was oriented perpendicular to the nearest sulcus and the coil was kept tangential to the head.

### Analysis of TMS

MEPs at rest were studied for latency and amplitude, active MEPs for latency. The first MEP was excluded since its amplitude may be significantly higher than that of the following MEPs (Brasil-Neto et al. 1994), and the mean of the following ten MEPs was calculated. The silent period durations were defined as the mean duration of absolute EMG silence from the end of the MEP to the return of the background EMG after excluding the longest and shortest response (Säisänen et al. 2008a, b).

In motor mappings, the MEPs with an amplitude of ≥ 50 µV were accepted as responses and the locations of stimuli were stored by the eXimia software as MRI coordinates. The centre-of-gravities (CoGs) (Wassermann et al. 1992) of the representation areas were determined using the stimulation site coordinates individually for each muscle as well as combined for the hand (APB, ADM and FDI) and arm (ECR, FCR and BB).

To enable the between-group comparison of the CoGs, the individual coordinates for the hand and arm CoGs and MRIs for each subject were spatially normalized to the standard space using the SPM8-software running on Matlab 7.4 (Mathworks Inc., Natick, MA, USA). To determine the within group variation between CoGs, ellipsoids of the 90% confidence interval were fitted to the CoG clusters by estimating the lengths and directions of the ellipsoid main axis based on Chi square distribution with Matlab (Niskanen et al. 2010).

The extents of the representation areas of hand (APB, ADM, FDI) and arm (ECR, FCR, BB) muscles were assessed using the spline interpolation method (Julkunen 2014), and the ratio of the extents of these representation areas was calculated. The overlap in the hand and arm representation areas was determined as a relative overlap (%): the area eliciting responses in both hand and arm muscles divided by the total area eliciting responses in either hand or arm muscles, or both.

### Analysis of MRI

VBM analysis was performed using the VBM8-toolbox (http://www.dbm.neuro.uni-jena.de/vbm/vbm8/) in SPM8 (Wellcome Department of Imaging Neuroscience, London; http://www.fil.ion.ucl.ac.uk/spm) running in Matlab R2007b. Template-O-Matic toolbox (Wilke et al. 2008) was used to create age-matching tissue probability maps. These maps were utilized in the DARTEL algorithm to create the final templates for normalization and segmentation (Ashburner 2007). The images were then segmented into GM, white matter (WM) and cerebrospinal fluid with an isotropic voxel size of 1.5mm^3^ and normalized into the standard space. Finally, the segments were smoothed with a 10 mm full-width at half maximum Gaussian kernel. Thereafter, whole brain voxel-wise analyses of the between-group differences in densities of GM and WM were performed. The brain regions showing a significant difference in GM or WM density were localized by a neuroradiologist. The nature of the whole brain analyses was exploratory and therefore, all significant (*p* < 0.001) clusters were included.

In addition, region-of-interest (ROI) analyses were conducted to evaluate the local GM densities within the motor and sensory cortices. Box-shaped ROIs (4 × 4 × 2 voxels) (6 mm in anterior-posterior, 6 mm in medial–lateral and 3 mm in superior-inferior direction) were manually placed in the anatomically defined face, hand and leg areas, separately for M1 and primary sensory cortices in both hemispheres (Supplementary Figure). This was the maximum size for the ROI to fit on the area, excluding WM as precisely as possible. ROI analyses were performed with subject-specific tissue probability maps for GM in Amide software (http://amide.sourceforge.net/). The result of this analysis (value between 0 and 1) represents the relative quantity and density of GM in ROI.

### Assessment of handedness, head circumference, manual dexterity and physical activity

Handedness was assessed using The Waterloo Handedness Questionnaire (revised and reduced form with 20 items) (Steenhuis et al. 1990). Head circumference was measured manually using the broadest part of the forehead, above the ears and at most prominent part of the back of the head. The box and block test (BBT) was used to assess motor speed and skill separately for both hands (Mathiowetz et al. 1985). The test score was the total number of wooden cubes (2 × 2 × 2 cm^3^) moved one at a time from one side of a box to the other in 1 min. Observations were made for any mirror movements while performing the test. In the analysis, for simplicity, the mean of the test scores performed with the right and left hand was used. Habitual physical activity was assessed by the PANIC Physical Activity Questionnaire (Haapala et al. 2014) administered by the parents with their child. Total physical activity was calculated in minutes per day by summing up the amounts of organized sports, organized exercise other than sports, unsupervised physical activity, physically active school transportation, physical activity during school recess and physical education.

### Statistical analysis

Differences between the groups (AS vs. controls) were analyzed using the unpaired Mann–Whitney U test and differences between the hemispheres (right and left) were analyzed using the paired Wilcoxon signed rank test. Correlations between motor representation areas, VBM ROI, manual dexterity and physical activity were analyzed using the Spearman’s rank correlation. Statistical analyses were performed with the SPSS statistical software, Version 22 (IBM Corporation, Somers, NY, USA). Differences with a *p*-value of ≤ 0.05 were considered statistically significant. In both whole brain and ROI VBM analyses, the groups were compared using the two-sample *t* test, with the uncorrected significance level set as 0.001.

## Results

Both the imaging and nTMS session were well-tolerated. No abnormalities related to motor areas were found in the MRIs. The demographic and clinical characteristics of the AS and control group are shown in Table 1. Although our original aim was to recruit only right-handed subjects, there was one left-handed and one ambidextrous (predominantly left-handed) boy in the AS group. The AS boys received lower scores in the BBT (right hand *p* = 0.028, left hand *p* = 0.003). The AS boys also performed more poorly with their left hand than with their right hand (*p* = 0.021), whereas no such difference was observed in the control group (*p* = 0.799). No mirror movements were observed during the BBT. The head circumference was smaller (*p* = 0.038) and habitual physical activity level was lower in AS subjects than in controls (*p* = 0.028). (Table 1).

**Table 1:** Demographics and clinical scores of the boys with AS (n = 8) and the healthy controls (n = 8), presented as mean (standard deviation)

	AS	Controls	p-value
Age	10 years 5 months (14 months)	10 years 5 months (11 months)	0.645
Body mass index	17.8 (1.9)	17.9 (1.7)	0.721
Height (cm)	141.1 (5.7)	142.4 (8.2)	0.645
Head circumference (cm)	53.6 (1.1)	55.3 (1.8)	0.038*
Waterloo handedness questionnaire (score)	14.4 (24.8)	32.0 (4.4)	0.030*
Manual dexterity, right hand (cubes/min)	**58 (7)**	65 (5)	0.028*
Manual dexterity, left hand (cubes/min)	**54 (5)**	64 (6)	0.003*
Physical activity (min/day)	67 (26)	115 (48)	0.028*
Ehlers–Gillberg (score)	32.5 (6.0)	1.5 (1.5)	< 0.001*
Autism spectrum quotient (score)	39 (5.2)	9.0 (7.0)	< 0.001*

The statistical significance of differences between groups is given as p-values (Mann–Whitney U Test). *P*-values ≤ 0.05 are marked with an asterisk. The AS boys also performed more poorly with the left hand than with the right hand (*p* = 0.021, Wilcoxon paired samples test, emboldened)

### nTMS

#### TMS parameters

None of the measured TMS parameters (rMT, MEP amplitude or latency, silent period duration) differed between the groups (Table 2). In one control subject, the rMT in the left hemisphere was 91% of maximum stimulator output, and thus, suprathreshold MEPs were stimulated using 110% instead of 120% of rMT. Hemispheric within-group asymmetry was observed in AS for silent period duration (shorter in the right hemisphere, *p* = 0.050) and for the active MEP latency (longer in the left hemisphere, *p* = 0.017). The control group exhibited hemispheric asymmetry for rMT (higher on the left hemisphere, *p* = 0.042).

**Table 2:** nTMS results presented as group means (standard deviation)

	Hemisphere	AS	Within-group	Control	Within-group	Between-groups
rMT (% of stimulator output)	L	56 (10)	0.944	62 (16)	0.042*	0.645
	R	56 (16)		58 (14)		0.721
Silent period duration (ms)	L	124 (30)	0.050*	117 (43)	0.499	0.867
	R	97 (34)		115 (39)		0.645
MEP at rest amplitude (mV)	L	0.60 (0.61)	0.889	0.73 (0.57)	0.735	0.161
	R	0.54 (0.34)		0.59 (0.18)		0.613
MEP at rest latency (ms)	L	20.6 (1.1)	0.674	21.0 (1.8)	0.686	0.382
	R	20.5 (1.3)		20.9 (1.7)		0.613
Active MEP latency (ms)	L	18.3 (1.4)	0.017*	18.3 (2.0)	0.611	0.878
	R	19.4 (2.0)		18.2 (1.8)		0.195
Area hand (cm^2^)	L	7.15 (3.30)	0.128	6.86 (4.12)	0.889	0.878
	R	5.76 (2.11)		6.51 (2.10)		0.867
Area arm (cm^2^)	L	8.59 (4.38)	0.176	6.36 (5.37)	0.484	0.234
	R	5.89 (4.07)		6.62 (2.84)		0.536
Overlap hand/arm (%)	L	60 (14)	0.043*	53 (13)	0.263	0.382
	R	53 (17)		57 (14)		0.955

Between-group differences were tested with Mann–Whitney U-test and within-group differences between the hemispheres with Wilcoxon signed rank test. Significant differences (p ≤ 0.05) are shown with asterisks

*rMT* resting motor threshold, *MEP* motor-evoked potential

#### Centre-of-gravities

**Fig. 1:**
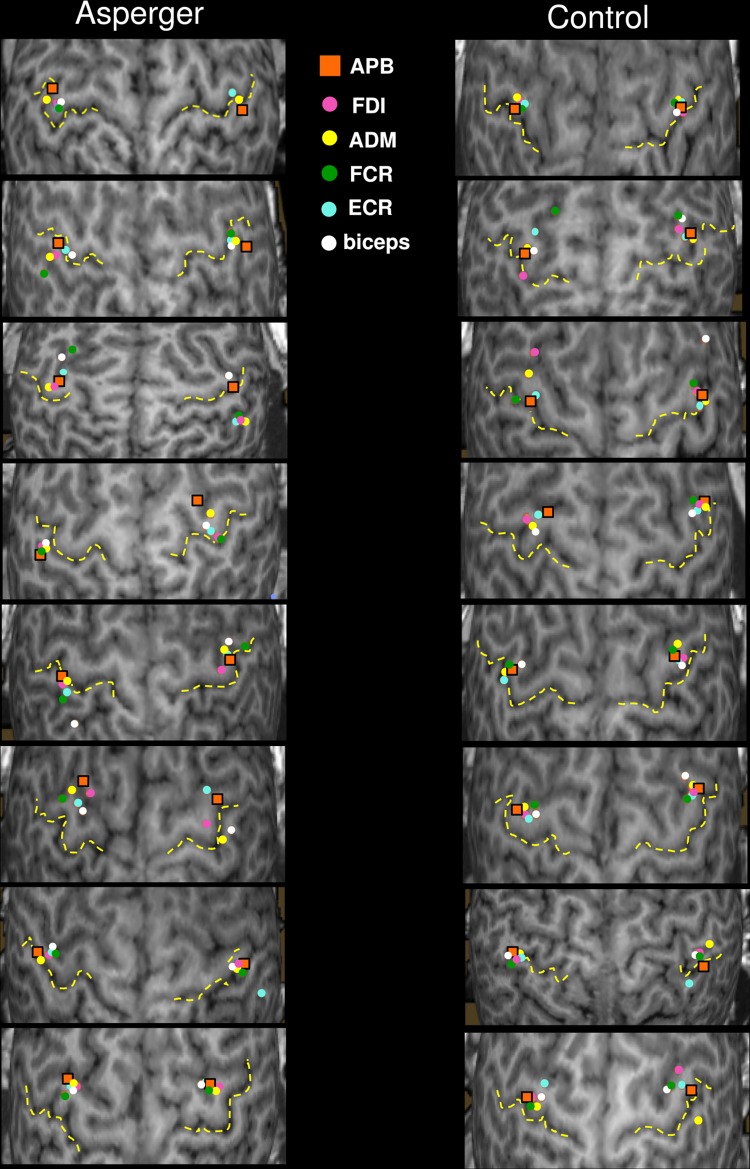
The individual muscle-specific centre-of-gravities (CoGs) in Asperger syndrome (AS) subjects (left panels) and controls (right panels). Central sulcus is shown with a dotted line. *APB* abductor pollicis brevis, *FDI* first dorsal interosseous, *ADM* adductor digiti minimi, *FCR* flexor carpi radialis, *ECR* extensor carpi radialis

**Fig. 2:**
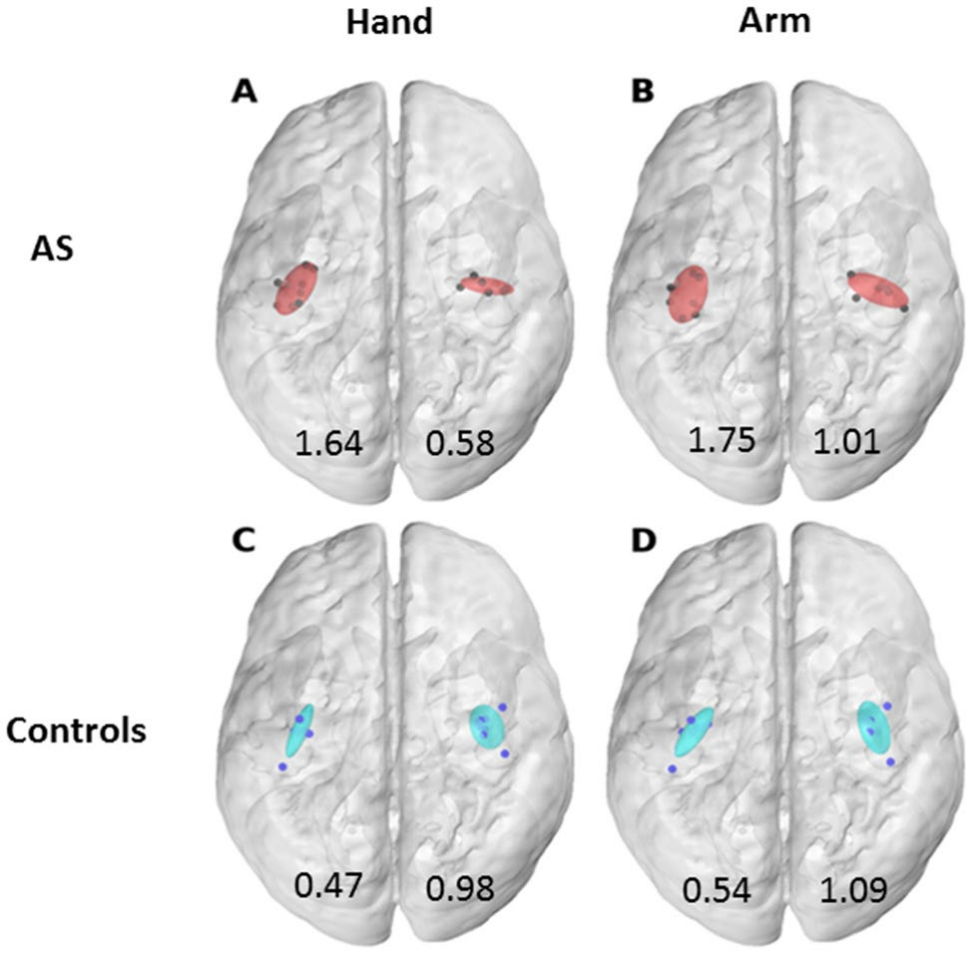
The locations of CoGs with 90% confidence interval in normalized standard brain and sizes of ellipsoids (cm^3^). **a** Asperger syndrome (AS)—hand CoGs, **b** AS—arm CoGs, **c** control—hand CoGs, **d** control—arm CoGs

The CoGs for each of the six muscles are presented for the individual 3D MRI brains in all subjects (Fig. 1). The visual inspection revealed that in healthy controls, the CoGs of single muscles were centered mainly in the expected hand knob area whereas in the AS boys, they were more widespread. In 7 out of 8 AS subjects, some of the muscle-specific CoGs were located in postcentral gyrus, or were located clearly anterior to the precentral gyrus (subjects #4, #5 and #8). A prominent interhemispheric asymmetry in the location of the main target muscle APB was observed in AS subjects #1 and #4. This was not observed in any of the control subjects. The normalized coordinates of CoGs for individual muscles, hand or arm did not differ between the groups. However, the CoGs were spatially less clustered in the AS group than in the control group: in the left hemisphere, the volumes of the 90% confidence interval ellipsoids were three times larger in the AS group and more round-shaped compared to the controls (Fig. 2). Furthermore, according to visual inspection, asymmetry was observed in the AS group, where the right hemisphere ellipsoid was oriented in the lateral-medial direction, compared to anterior-posterior orientation in the left hemisphere.

#### Representation areas

**Fig. 3:**
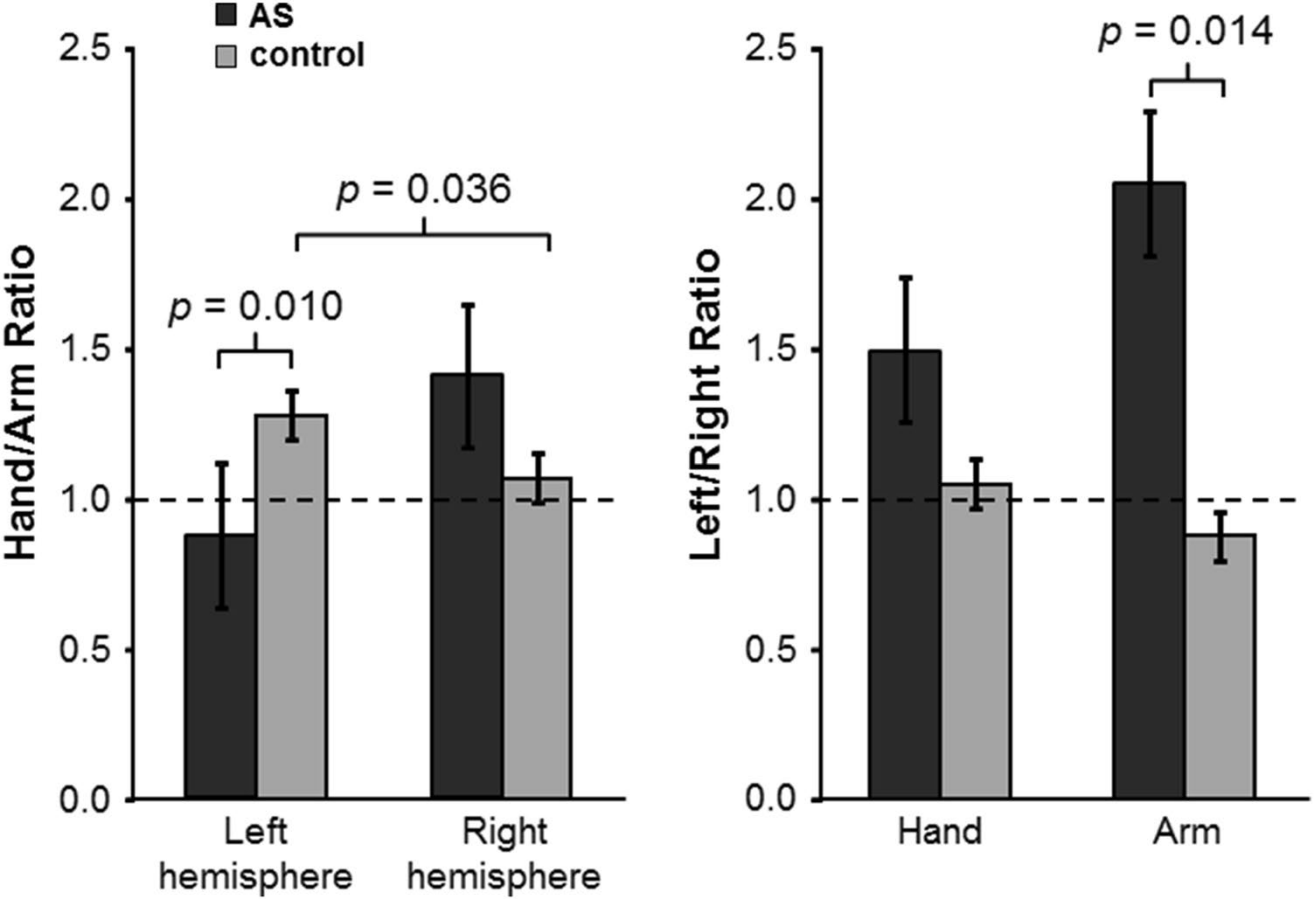
Ratios of representation areas, hand/arm, left/right (mean ± standard error of the mean) in AS and controls. Statistically significant differences are indicated with *p*-values

The absolute representation areas of hand or arm displayed no significant differences between the groups (Table 2). However, there was a tendency towards interhemispheric asymmetry (larger representation areas in the left hemisphere for both hand and arm) in the AS boys whereas the left/right ratio in the controls was around one. When the representation ratios (hand area / arm area) were examined, the controls showed an interhemispheric difference (*p* = 0.036): larger hand than arm representation in the left, dominant hemisphere and a ratio of approximately 1 in the right hemisphere (Fig. 3). Instead, the AS subjects exhibited a different pattern and extensive variability (*p* = 0.398): in the left hemisphere, the arm was represented on a larger area than the hand, whereas in the right hemisphere, the hand was represented on a larger area than the arm (Fig. 3). The overlap of hand and arm representations did not differ between the groups, but an interhemispheric difference (a larger overlap in the left hemisphere) was observed in the AS subjects (*p* = 0.043) (Table 2).

#### Correlations between representation area ratios, manual dexterity and physical activity

In the combined group of both AS and controls, the ratios of representation areas correlated with manual dexterity: hand/arm ratio in the left hemisphere correlated positively with BBT score (*rho* = 0.528, *p* = 0.036) and left/right ratio for arm correlated negatively with BBT score (*rho* = − 0.760, *p* = 0.001) (Table 3). When only the AS group was considered, the hand/arm ratio in the right hemisphere correlated negatively with the BBT score (*rho* = − 0.893, *p* = 0.007). In controls only, the left/right ratio for arm correlated negatively with the BBT score (*rho* = − 0.731, *p* = 0.040). The BBT score also correlated with habitual physical activity level.

**Table 3:** Correlations (Spearman’s rho) between motor mapping with nTMS, imaging (region-of-interest) data from voxel-based morphometry analysis, manual dexterity and physical activity

BBT	Motor mapping				Imaging data		Physical activity
	Left/right ratio		Hand/arm ratio		Mean motor	Mean sensor	
	Hand	Arm	Left	Right			
All	− 0.301 (*p* = 0.276)	− 0.760* (*p* = 0.001)	0.528* (*p* = 0.036)	− 0.272 (*p* = 0.326)	− 0.407 (*p* = 0.118)	− 0.006 (*p* = 0.983)	0.723* (*p* = 0.002)
AS	0.464 (*p* = 0.294)	− 0.607 (*p* = 0.148)	− 0.143 (*p* = 0.736)	− 0.893* (*p* = 0.007)	0.119 (*p* = 0.779)	0.881* (*p* = 0.004)	0.524 (*p* = 0.183)
Controls	− 0.515 (*p* = 0.192)	− 0.731* (*p* = 0.040)	0.455 (*p* = 0.257)	0.156 (*p* = 0.713)	− 0.719* (*p* = 0.045)	− 0.359 (*p* = 0.382)	0.443 (*p* = 0.272)
Between-groups	*p* = 0.232	*p* = 0.014*	*p* = 0.010*	*p* = 0.755	*p* = 0.065	*p* = 0.574	*p* = 0.028*

The sign for correlations sometimes differs between the groups. Significant correlations (*p* ≤ 0.05) are indicated with asterisk

### MRI

#### Whole brain analysis

**Fig. 4:**
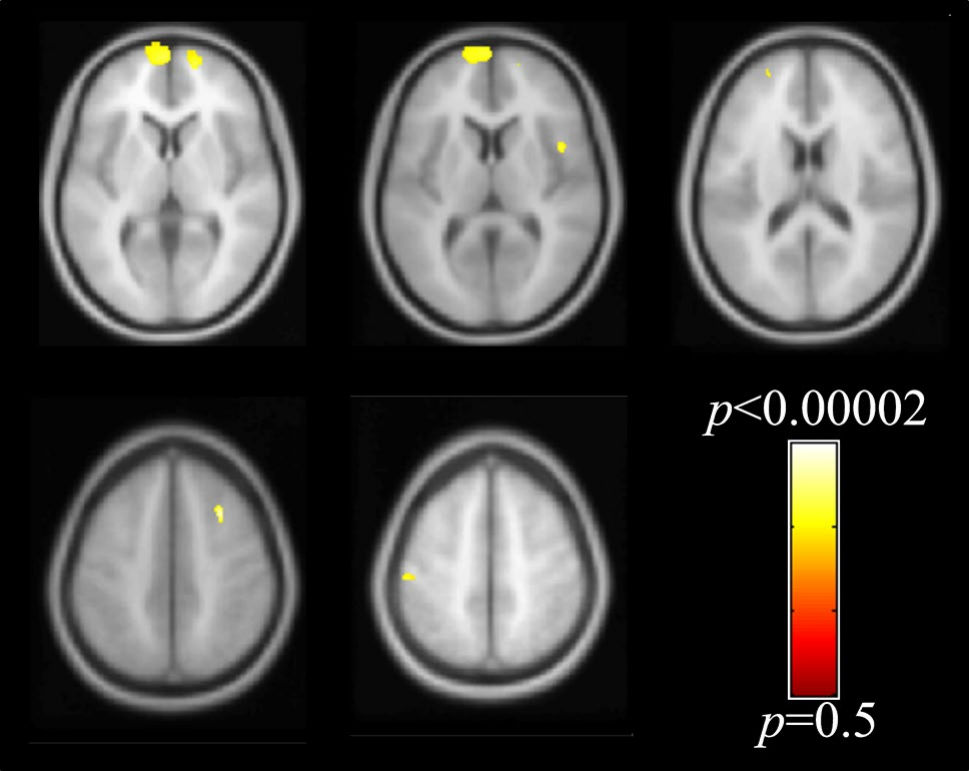
Voxel-wise whole brain voxel-based morphometry results of the GM shown with respect to the neurologic orientation (*p* < 0.001, uncorrected). Areas with decreased GM density in AS as compared to controls are shown in representative axial slices

**Fig. 5:**
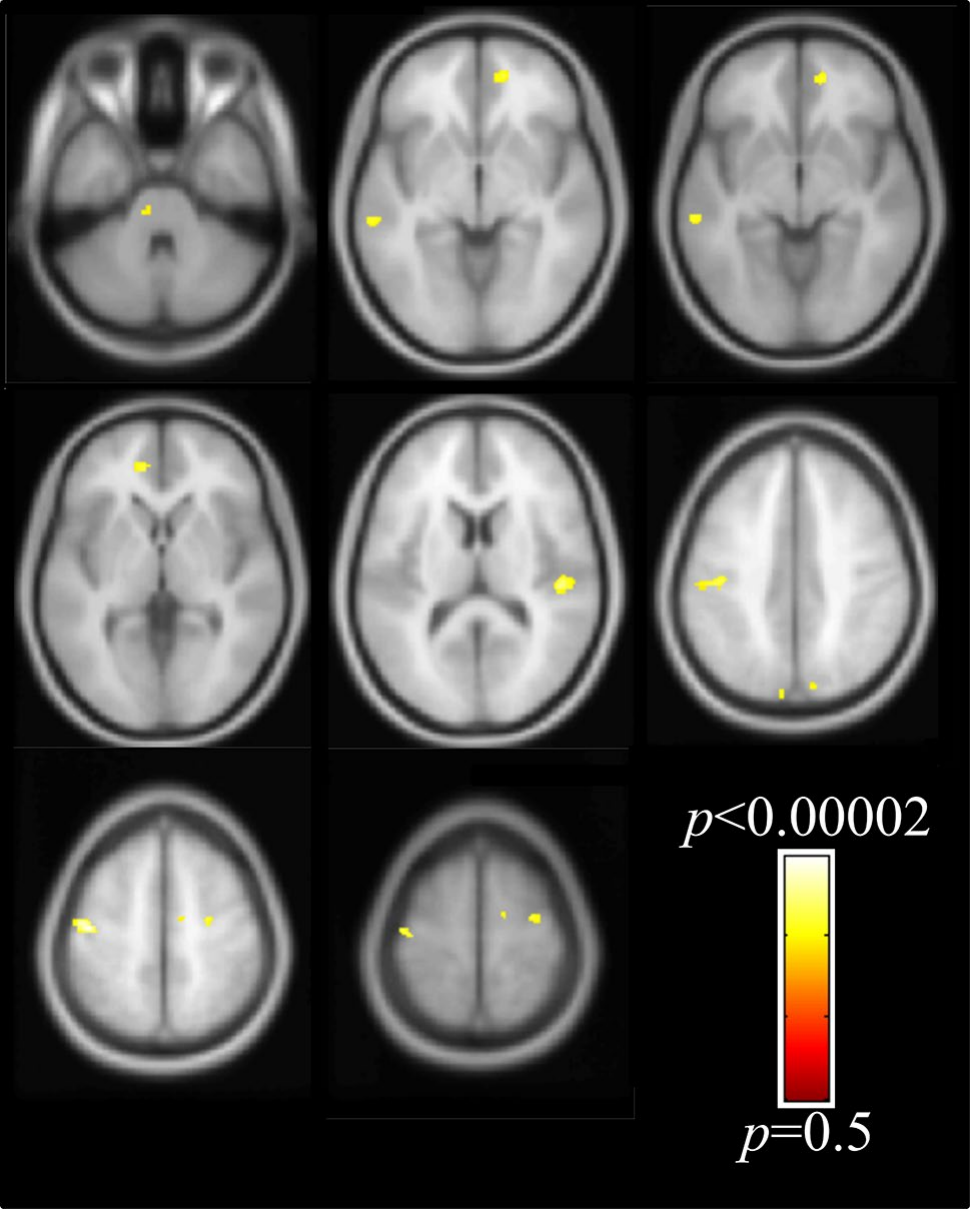
Voxel-wise whole brain voxel-based morphometry results of the WM shown with respect to the neurologic orientation (*p* < 0.001, uncorrected). Areas with decreased WM density in AS as compared to controls are shown in representative axial slices. The findings are seen on gray matter since they are shown in average template

Differences between groups were found as small areas of decreased densities of GM in AS in comparison to controls (Fig. 4**)**. One small area of decreased density was located in the sensorimotor area, specifically in the left postcentral gyrus. Additional small areas of decreased densities in GM were found bilaterally in the superior frontal gyri, medial frontal gyri and inferior frontal gyrus (pars opercularis). Small areas of decreased WM density in AS were found in the bilateral precentral gyrus (Fig. 5). Additional areas with decreased WM density were detected in bilateral superior frontal gyri, superior occipital/parietal gyrus, left superior temporal gyrus and right medial temporal gyrus (*p*-value ≤ 0.001, uncorrected). No areas of increased GM or WM in AS versus controls were observed.

#### ROI analysis

In ROI analysis, differences between groups were found in the left hemisphere face sensory area, showing reduced GM density in AS (*p* = 0.050), and in right hemisphere leg motor area, showing increased GM density in AS (*p* = 0.050). A hemispheric difference was found in AS in the face sensory area, *i.e*. there was less GM in the left hemisphere (*p* = 0.012). The controls did not exhibit hemispheric differences, although they did exhibit a trend towards more GM on the left M1 and primary sensory cortex (*p* = 0.161) whereas the AS group did not display any such trend.

#### Correlations between GM, manual dexterity and physical activity

When all subjects (AS and controls) were considered, the BBT score correlated negatively with GM in the left hand motor cortex (*rho* = − 0.602, *p* = 0.014). In controls, the left hand motor cortex showed a trend (*rho* = − 0.683, *p* = 0.062), and left leg motor cortex revealed a significant negative correlation with the BBT score (*rho* = − 0.874, *p* = 0.005). In the AS group, none of the motor areas correlated with the BBT score, but instead, right hand sensory cortex correlated *positively* with the BBT score (*rho* = 0.762, *p* = 0.028). This is also reflected in the mean motor and mean sensory GM correlations with BBT score (Table 3). There was also a trend towards a between-group difference in the mean motor GM (*p* = 0.065) (Table 3). *Physical activity*, in all subjects, correlated negatively with GM in the left hand (*rho* = − 0.655, *p* = 0.006) and leg motor cortices (*rho* = − 0.503, *p* = 0.047).

## Discussion

In this study, we found functional reorganization of motor cortex in pediatric AS population having fine motor impairment with nTMS with concomitant abnormalities in structural MRI. Observed structural and functional alterations correlated with the behavioral evaluation of manual dexterity. Due to the small number of subjects, this trial needs to be considered as a proof-of-concept study. This was, however, the first time that nTMS mapping has been utilized in this patient population.

Aberrant, i.e. less focused and anatomically more variable, motor cortical organization was observed in the AS children as compared to the controls. Functional asymmetry was found in the AS subjects who displayed a larger representation area in the left hemisphere as compared to the right hemisphere. The distribution (i.e. ratios of representation areas) of hand and arm muscles was also different, i.e. there was a more pronounced arm representation in the left hemisphere and the opposite pattern in the right hemisphere. The overlap of hand and arm representations also revealed an interhemispheric difference (a larger overlap in the left hemisphere) in AS subjects. These functional aberrations were supported by exploratory structural imaging methods that demonstrated small areas of reduced GM in the left and reduced WM densities in bilateral sensorimotor areas in AS children compared to controls. The observed functional and structural aberrations were associated with impaired motor skills. These alterations are consistent with the clinical picture, and may underlie the manual dexterity impairment often encountered in children with AS.

The characteristics of motor function measured with TMS did not differ between AS and controls. However, asymmetry between the hemispheres was observed in AS in the form of weaker inhibition (shorter silent period duration) in the right hemisphere as compared with the left; this kind of asymmetry is not observed in normal development (Säisänen et al. 2018). There was also an interhemispheric asymmetry in AS for the active MEP latency, which was prolonged in the left hemisphere. In a previous study, diffusion imaging detected differences between ASD and controls in the left hemisphere that could be associated with the reductions in WM tract coherence and organization, myelination and conduction speed (Thompson et al. 2017). This might explain the hemispheric latency differences. Previously, laterality effects in TMS-measures have not been observed in ASD (Enticott et al. 2010), with the exception of an SICI deficit in the left hemisphere (Enticott et al. 2013). These authors also observed a longer silent period duration in ASD subjects without any language-delay when they were compared to children experiencing a language-delay (which was similar to controls) (Enticott et al. 2013).

The motor mappings revealed a less focused organization in AS children in comparison to controls. In seven out of eight boys with AS, the functional hand motor areas were located partly outside the primary motor cortex, usually involving the postcentral gyrus and/or anterior to the precentral gyrus (premotor area). Motor functions located partly in sensory cortex can reflect that the sensory cortex has taken over functions of motor cortex, or it can be related to impaired sensorimotor integration. Somewhat similar findings have been observed in hemiplegic cerebral palsy where abnormal somatotopic organization of functional primary sensory cortex to the precentral sulcus was observed (Papadelis et al. 2018). This finding of functional reorganization was also associated with a structural finding using diffusion tractography, pointing out the importance of multimodal imaging, and also correlated with behaviorally-assessed sensory deficits. On the other hand, in a study comparing children with ASD with children with developmental coordination disorder, sensory input appeared unaffected in ASD (Paquet et al. 2019). There is profound interconnectedness of the sensorimotor system as reviewed by Hooks (2017). Though these disorders are different, they share similarities in motor functioning (Bhat et al. 2011). However, based on the current or the study by Papadelis and co-workers, it cannot be concluded whether these changes are adaptive or maladaptive.

In general, when located with TMS, the individual muscles are usually represented in primary motor cortex whereas the preparation and control of complex movement sequences and coarse movements are located in the premotor and supplementary motor areas (Shibasaki 2012). A larger spread in the anatomical variation of the representations of the hand and arm CoGs in the left hemisphere was also evident in the AS subjects in comparison with the controls. This finding is in line with previous imaging studies exploiting other modalities. A functional MRI (fMRI) study found that in individuals with autism, there were several types of atypical activation patterns, often with an unusual spread of activations into prefrontal and superior parietal regions, whereas in controls, the strongest motor activations were consistently along the contralateral central sulcus and included also supplementary motor areas (Müller et al. 2001). Another fMRI study detected decreased connectivity across the whole motor execution network and greater activation in supplementary motor areas in children with high-functioning autism (Mostofsky et al. 2009). A magnetoencephalography study revealed a deviating relative localization between the thumb and the index finger in the left hemisphere in young adults with ASD (Coskun et al. 2009). Evidence of structurally and functionally altered cortical area formation during development has been found in a mouse model of ASD (Fenlon et al. 2015). There is also a preliminary study using TMS-EEG conducted in adults with ASD, which found no differences between the electrophysiological responses to TMS, but there was a trend toward reduced phase synchrony in the primary motor cortex beta band correlating with several clinical features (Kirkovski et al. 2016).

We found larger upper extremity muscle representation areas in the left hemisphere as compared to the right in the AS subjects (statistically significant for arm, tendency for hand), whereas in controls, they were rather symmetric. In healthy adults, a larger motor representation in the dominant hemisphere has been observed (Triggs et al. 1999; Chieffo et al. 2016) though motor areas of equal size have also been reported (Schwenkreis et al. 2007). This issue remains controversial. Moreover, in the left hemisphere, the representation area of the arm was larger than that of the hand in AS subjects, whereas in controls, the representation area of the hand was larger than that of the arm. In the right hemisphere, on the contrary, the extents of hand and arm representations were similar in controls, whereas the AS subjects exhibited larger hand than arm representations. The overrepresentation of arm muscles in the left hemisphere and hand muscles in the right hemisphere may be related to the pathophysiology of impaired motor function or could reflect the capacity for skilled learning, although the functional relevance of this finding is not known. There is sensorimotor cortical representation reorganization in professional musicians related to task-specific practice (Schwenkreis et al. 2007; Vaalto et al. 2013; Chieffo et al. 2016). Evidence from animal studies has indicated that a greater proportion of distal representations and a smaller proportion of those on the proximal side are related to the late phase of motor learning and cortical plasticity (Kleim et al. 2004). One study applying a multimodal imaging approach also revealed reversed asymmetry and an unusual degree of right hemisphere motor participation in ASD (Carper et al. 2015).

The overlap of arm and hand representation areas did not differ between groups, but evidence of interhemispheric asymmetry (more overlap in the left hemisphere) was observed in the AS subjects. In healthy adults, the muscle representation areas of hand, wrist and shoulder have been shown to overlap substantially and to be of equal size even though the locations of their optimal sites or CoGs can be distinguished (Devanne et al. 2006; Marconi et al. 2007; Melgari et al. 2008). Further clarifications of the somatotopical or core-surround organization as well as a better representation of synergistic movements have been provided by fMRI (Strother et al. 2012). Map characteristics and differences in overlap also vary according to the task being performed (Masse-Alarie et al. 2017). Increased overlap between the proximal and distal representations has been found in professional sportswomen, this being attributable to extensive training (Tyc et al. 2005) or in chronic pain (Schabrun et al. 2014). However, the significance of the overlap or its development during maturation is not fully clear.

Anatomically, we found concomitant decreases in the amounts of GM and WM in the left sensorimotor cortex in AS. This is in line with previous studies (DeRamus and Kana 2015) and the scarcity of GM was reflected in the nTMS mapping result of aberrant somatotopy (less focused representation areas). Due to the spatial error in individual functional areas, when the results are visualized in average template, we further examined the brain areas by individual ROIs, which resulted in differences between the groups in the left hemisphere face sensory area (reduced GM in AS) and the right hemisphere leg motor area (increased GM in AS). In addition, a hemispheric difference was found in the AS group i.e. they had less GM in the left face sensory area. The controls did not exhibit hemispheric differences, but rather showed a trend towards more GM in the left primary sensorimotor areas, a trend not evident in the AS group. Duffield and coworkers evaluated ASD subjects; there were no gross volumetric differences found in key motor areas, but the volume of the precentral gyrus negatively correlated with the finger tapping test speed (Duffield et al. 2013). This was interpreted according to overgrowth theory of autism where a larger size of motor cortex is associated with poorer function or less efficient networks (Duffield et al. 2013).

When all subjects were analyzed together, the relatively larger hand and smaller arm representation in the left, dominant hemisphere correlated with better dexterity. In AS, the larger hand than arm representation in the right hemisphere correlated strongly and negatively with poorer dexterity. This is in line with one report where correlations were found with motor performance in the right hemisphere in ASD, whereas in controls who displayed a typical left dominance, the correlations with motor skills were present in the left hemisphere (Thompson et al. 2017). The same study revealed abnormalities in several DTI measures in ASD in the left hemisphere. Manual dexterity correlated negatively with the amount of GM in the left motor cortex in controls which can be expected since GM decreases with age as the motor functions improve (Giedd et al. 2009). When different motor tasks were compared, the finger tapping test was the only one to correlate negatively with precentral gyrus motor ROI volume in ASD (Duffield et al. 2013). Instead, in AS, the hand sensory cortex in the right hemisphere showed a positive correlation with the BBT score and it can be speculated that some of the motor functions are being taken over by the sensory cortex in AS.

There is a large amount of scientific studies on motor dysfunction in ASD, and standardized neuro-psychomotor assessments can be used (Paquet et al. 2016). As well, neurological soft signs can be evaluated in the context of different childhood neuropsychiatric disorders (D’Agati et al. 2018; Pitzianti et al. 2016). Our result of the difference between cortical representation areas for hand and arm, can be considered to fit well with the previous description of hypertonia in proximal muscles accompanied by hypotonia in distal muscles, (Paquet et al. 2016). There were lateralization disturbances only in ASD, when compared to developmental coordination disorder, which supported the hypothesis of proprioceptive impairment due to visual fixation problems influenced by muscular tone in relation to the subcortical and cortical structures and possible interhemispheric disorder (Paquet et al. 2019). We also found interhemispheric differences in ASD (manual dexterity, silent period duration, active MEP latency and motor maps), though our findings need to be considered as preliminary, and this exploratory study unfortunately cannot enlighten the origins of the deficits better.

Similar correlations were observed with respect to physical activity, which in turn correlated with manual dexterity. Interestingly, the groups differed significantly in terms of their habitual physical activity levels: AS subjects only took about half as much daily physical activity as controls. This same positive correlation between physical activity and manual dexterity has also been previously shown in adults with AS (Sahlander et al. 2008). It is possible that the factors underlying the motor impairment are related to less experience of physical activity as well as poorer motivation in AS subjects (Duffield et al. 2013). Activity and experience, or other factors including social activities involving an active lifestyle, support the organization of functional networks in typical brain development, and all of these can be impaired in AS. In ASD there is a deficit in visual compensation (Paquet et al. 2019), which in tasks with high proprioceptive component such as imitation of movement, balance and walking, may limit the amount of physical activity.

### Strengths and limitations of the study

This study applied novel methodologies (neuronavigation and motor mapping) for studying functional characteristics in ASD. The aiming tool in the navigation system allowed us to assess the TMS measures with good accuracy. Despite our attempts to recruit all eligible subjects with AS in the Northern Savo Hospital District, due to its low number of participants, this study may be underpowered to detect subtle differences between groups, and thus should be considered mainly as proof-of-concept trial with preliminary results. One strength of the study is the homogenous population being evaluated. None of the subjects was receiving any medication; the subjects were preadolescents with a narrow age-span and strict age-matching with controls was applied, which means that one does not have to consider the complicated question of age-dependency. Methodologically, DTI is more accurate than VBM for examining WM (Brito et al. 2009; Kumar et al. 2010) and in that respect, our WM results need to be viewed as estimates. Generally, hand preference in ASD differs from the general population with a marked overrepresentation of left-handedness and inconsistent, mixed handedness (Lindell and Hudry 2013). The mixed hand dominance of the subjects might theoretically have affected the results, especially in such a small group. It would also have been desirable to undertake several tests to assess different aspects of manual dexterity (Duffield et al. 2013; Paquet et al. 2016). The larger motor maps of the AS were not affected by the head size, since in the current study population, the head circumference was smaller in the AS group than in the control group, in contrast to earlier observations (Redcay and Courchesne 2005). Finally, it might have been advantageous to examine several more proximal muscles such as triceps and anterior deltoid, in order to reveal other potential differences in the motor maps (Plow et al. 2014).

In conclusion, we applied MRI-navigated TMS to map the motor representation areas of the upper limb in AS subjects. The functional aberrations detected were corroborated by MR imaging findings and they also correlated with the manual dexterity of the trial participants.

## Electronic supplementary material

Below is the link to the electronic supplementary material.

Supplementary Figure—On the left, region-of interests (ROIs) of the leg, hand and face areas in both M1 (red) and primary sensory cortex (blue) from a representative subject. On the right, the level where the ROIs were placed in the coronal plane. Footnote: Of note. The ROIs cover several slices and the center of the ROI was not in the same slice in any of the subject. Thereby, as the aim of this figure was to visualize all ROIs in the same slice, the face ROI seems to be in WM, which is not the case. (TIF 1474 KB)

## References

[CR1] Ashburner J (2007). A fast diffeomorphic image registration algorithm. NeuroImage.

[CR2] Auyeung B, Baron-Cohen S, Wheelwright S, Allison C (2008). The autism spectrum quotient: children’s version (AQ-Child). J Autism Dev Disord.

[CR3] Awiszus F (2003). TMS and threshold hunting. Suppl Clin Neurophysiol.

[CR4] Awiszus F, Borckardt J (2012) TMS motor threshold assessment tool (MTAT 2.0). http://clinicalresearcher.org/software.htm. Accessed 16 October 2017

[CR5] Barbeau EB, Meilleur AA, Zeffiro TA, Mottron L (2015). Comparing motor skills in autism spectrum individuals with and without speech delay. Autism Res.

[CR6] Behere A, Shahani L, Noggle CA, Dean R (2012). Motor functioning in autistic spectrum disorders: a preliminary analysis. J Neuropsychiatry Clin Neurosci.

[CR7] Bhat AN, Landa RJ, Galloway JC (2011). Current perspectives on motor functioning in infants, children, and adults with autism spectrum disorders. Phys Therapy.

[CR8] Brasil-Neto JP, Cohen LG, Hallett M (1994). Central fatigue as revealed by postexercise decrement of motor evoked potentials. Muscle Nerve.

[CR9] Brito AR, Vasconcelos MM, Domingues RC, Hygino da Cruz LC, Rodrigues Lde S, Gasparetto EL, Calcada CA (2009). Diffusion tensor imaging findings in school-aged autistic children. J Neuroimaging.

[CR10] Carper RA, Solders S, Treiber JM, Fishman I, Muller RA (2015). Corticospinal tract anatomy and functional connectivity of primary motor cortex in autism. J Am Acad Child Adolesc Psychiatry.

[CR11] Cauda F, Geda E, Sacco K, D’Agata F, Duca S, Geminiani G, Keller R (2011). Grey matter abnormality in autism spectrum disorder: an activation likelihood estimation meta-analysis study. J Neurol Neurosurg Psychiatry.

[CR12] Chieffo R, Straffi L, Inuggi A, Gonzalez-Rosa JJ, Spagnolo F, Coppi E, Nuara A, Houdayer E, Comi G, Leocani L (2016). Motor cortical plasticity to training started in childhood: the example of piano players. PLoS ONE.

[CR86] Conde V, Vollmann H, Sehm B, Taubert M, Villringer A, Ragert P (2012). Cortical thickness in primary sensorimotor cortex influences the effectiveness of paired associative stimulation. NeuroImage..

[CR13] Coskun MA, Varghese L, Reddoch S, Castillo EM, Pearson DA, Loveland KA, Papanicolaou AC, Sheth BR (2009). How somatic cortical maps differ in autistic and typical brains. Neuroreport.

[CR14] D’Agati E, Pitzianti M, Curatolo P, Pasini A (2018). Scientific evidence for the evaluation of neurological soft signs as atypical neurodevelopmental markers in childhood neuropsychiatric disorders. J Psychiatr Pract.

[CR87] Dayan E, López-Alonso V, Liew SL, Cohen LG (2018). Distributed cortical structural properties contribute to motor cortical excitability and inhibition. Brain Struct Funct.

[CR15] DeRamus TP, Kana RK (2015). Anatomical likelihood estimation meta-analysis of grey and white matter anomalies in autism spectrum disorders. NeuroImage-Clin.

[CR16] Devanne H, Cassim F, Ethier C, Brizzi L, Thevenon A, Capaday C (2006). The comparable size and overlapping nature of upper limb distal and proximal muscle representations in the human motor cortex. Eur J Neurosci.

[CR17] Duffield TC, Trontel HG, Bigler ED, Froehlich A, Prigge MB, Travers B, Green RR, Cariello AN, Cooperrider J, Nielsen J, Alexander A, Anderson J, Fletcher PT, Lange N, Zielinski B, Lainhart J (2013). Neuropsychological investigation of motor impairments in autism. J Clin Exp Neuropsychol.

[CR18] Ehlers S, Gillberg C, Wing L (1999). A screening questionnaire for Asperger syndrome and other high-functioning autism spectrum disorders in school age children. J Autism Dev Disord.

[CR19] Eloranta AM, Lindi V, Schwab U, Tompuri T, Kiiskinen S, Lakka HM, Laitinen T, Lakka TA (2012). Dietary factors associated with overweight and body adiposity in Finnish children aged 6–8 years: the PANIC Study. Int J Obes (2005).

[CR20] Enticott PG, Rinehart NJ, Tonge BJ, Bradshaw JL, Fitzgerald PB (2010). A preliminary transcranial magnetic stimulation study of cortical inhibition and excitability in high-functioning autism and Asperger disorder. Dev Med Child Neurol.

[CR21] Enticott PG, Kennedy HA, Rinehart NJ, Tonge BJ, Bradshaw JL, Fitzgerald PB (2013). GABAergic activity in autism spectrum disorders: an investigation of cortical inhibition via transcranial magnetic stimulation. Neuropharmacology.

[CR22] Fenlon LR, Liu S, Gobius I, Kurniawan ND, Murphy S, Moldrich RX, Richards LJ (2015). Formation of functional areas in the cerebral cortex is disrupted in a mouse model of autism spectrum disorder. Neural Dev.

[CR23] Floris DL, Barber AD, Nebel MB, Martinelli M, Lai M-C, Crocetti D, Baron-Cohen S, Suckling J, Pekar JJ, Mostofsky SH (2016). Atypical lateralization of motor circuit functional connectivity in children with autism is associated with motor deficits. Mol Autism.

[CR24] Frye RE, Rotenberg A, Ousley M, Pascual-Leone A (2008). Transcranial magnetic stimulation in child neurology: current and future directions. J Child Neurol.

[CR25] Garvey MA, Ziemann U, Bartko JJ, Denckla MB, Barker CA, Wassermann EM (2003). Cortical correlates of neuromotor development in healthy children. Clin Neurophys.

[CR26] Giedd JN, Lalonde FM, Celano MJ, White SL, Wallace GL, Lee NR, Lenroot RK (2009). Anatomical brain magnetic resonance imaging of typically developing children and adolescents. J Am Acad Child Adolesc Psychiatry.

[CR27] Gowen E, Hamilton A (2013). Motor abilities in autism: a review using a computational context. J Autism Dev Disord.

[CR28] Haapala EA, Poikkeus AM, Kukkonen-Harjula K, Tompuri T, Lintu N, Väistö J, Leppänen PH, Laaksonen DE, Lindi V, Lakka TA (2014). Associations of physical activity and sedentary behavior with academic skills-a follow-up study among primary school children. PLoS ONE.

[CR29] Hooks BM (2017). Sensorimotor convergence in circuitry of the motor cortex. Neuroscientist.

[CR30] Julkunen P (2014). Methods for estimating cortical motor representation size and location in navigated transcranial magnetic stimulation. J Neurosci Methods.

[CR31] Julkunen P, Säisänen L, Danner N, Niskanen E, Hukkanen T, Mervaala E, Könönen M (2009). Comparison of navigated and non-navigated transcranial magnetic stimulation for motor cortex mapping, motor threshold and motor evoked potentials. NeuroImage.

[CR32] Jung NH, Janzarik WG, Delvendahl I, Münchau A, Biscaldi M, Mainberger F, Bäumer T, Rauh T, Mall V (2013). Impaired induction of long-term potentiation-like plasticity in patients with high-functioning autism and Asperger syndrome. Dev Med Child Neurol.

[CR33] Kallioniemi E, Julkunen P (2016). Alternative stimulation intensities for mapping cortical motor area with navigated TMS. Brain Topogr.

[CR34] Kaye HL, Rotenberg A, Krieg SM (2017). nTMS in pediatrics: special issues and solutions. Navigated transcranial magnetic stimulation in neurosurgery.

[CR88] Kearney-Ramos TE, Lench DH, Hoffman M, Correia B, Dowdle LT, Hanlon CA (2018). Gray and white matter integrity influence TMS signal propagation: a multimodal evaluation in cocaine-dependent individuals. Sci Rep.

[CR35] Kirkovski M, Rogasch NC, Saeki T, Fitzgibbon BM, Enticott PG, Fitzgerald PB (2016). Single pulse transcranial magnetic stimulation-electroencephalogram reveals no electrophysiological abnormality in adults with high-functioning autism spectrum disorder. J Child Adolesc Psychopharmacol.

[CR36] Kleim JA, Hogg TM, VandenBerg PM, Cooper NR, Bruneau R, Remple M (2004). Cortical synaptogenesis and motor map reorganization occur during late, but not early, phase of motor skill learning. J Neurosci.

[CR37] Krieg SM, Sabih J, Bulubasova L, Obermueller T, Negwer C, Janssen I, Shiban E, Meyer B, Ringel F (2014). Preoperative motor mapping by navigated transcranial magnetic brain stimulation improves outcome for motor eloquent lesions. Neuro-Oncology.

[CR38] Kumar A, Sundaram SK, Sivaswamy L, Behen ME, Makki MI, Ager J, Janisse J, Chugani HT, Chugani DC (2010). Alterations in frontal lobe tracts and corpus callosum in young children with autism spectrum disorder. Cereb Cortex.

[CR39] Lin K-L, Pascual-Leone A (2002). Transcranial magnetic stimulation and its applications in children. Chang Gung Med J.

[CR40] Lindell AK, Hudry K (2013). Atypicalities in cortical structure, handedness, and functional lateralization for language in autism spectrum disorders. Neuropsychol Rev.

[CR41] Mahajan R, Dirlikov B, Crocetti D, Mostofsky SH (2016). Motor circuit anatomy in children with autism spectrum disorder with or without attention deficit hyperactivity disorder. Autism Res.

[CR42] Marconi B, Pecchioli C, Koch G, Caltagirone C (2007). Functional overlap between hand and forearm motor cortical representations during motor cognitive tasks. Clin Neurophysiol.

[CR43] Masse-Alarie H, Bergin MJG, Schneider C, Schabrun S, Hodges PW (2017). “Discrete peaks” of excitability and map overlap reveal task-specific organization of primary motor cortex for control of human forearm muscles. Hum Brain Mapp.

[CR44] Mathiowetz V, Federman S, Wiemer D (1985). Box and block test of manual dexterity: norms for 6–19 year olds. Can J Occup Therapy.

[CR45] Melgari J-M, Pasqualetti P, Pauri F, Rossini PM (2008). Muscles in “concert”: study of primary motor cortex upper limb functional topography. PLoS ONE.

[CR46] Mostofsky SH, Powell SK, Simmonds DJ, Goldberg MC, Caffo B, Pekar JJ (2009). Decreased connectivity and cerebellar activity in autism during motor task performance. Brain.

[CR47] Müller K, Kass-Iliyya F, Reitz M (1997). Ontogeny of ipsilateral corticospinal projections: a developmental study with transcranial magnetic stimulation. Ann Neurol.

[CR48] Müller R-A, Pierce K, Ambrose JB, Allen G, Courchesne E (2001). Atypical patterns of cerebral motor activation in autism: a functional magnetic resonance study. Biol Psychiatry.

[CR49] Nezu A, Kimura S, Kobayashi T, Tanaka M, Saito K (1997). Magnetic stimulation of motor cortex in children: maturity of corticospinal pathway and problem of clinical application. Brain Dev.

[CR50] Nickl-Jockschat T, Habel U, Michel TM, Manning J, Laird AR, Fox PT, Schneider F, Eickhoff SB (2012). Brain structure anomalies in autism spectrum disorder-a meta-analysis of VBM studies using anatomic likelihood estimation. Hum Brain Mapp.

[CR51] Niskanen E, Julkunen P, Säisänen L, Vanninen R, Karjalainen P, Könönen M (2010). Group-level variations in motor representation areas of thenar and anterior tibial muscles: navigated transcranial magnetic stimulation study. Hum Brain Mapp.

[CR52] Nudo RJ, Wise BM, SiFuentes F, Milliken GW (1996). Neural substrates for the effects of rehabilitative training on motor recovery after ischemic infarct. Science.

[CR53] Oberman LM, Rotenberg A, Pascual-Leone A (2015). Use of transcranial magnetic stimulation in autism spectrum disorders. J Autism Dev Disord.

[CR54] Papadelis C, Butler EE, Rubenstein M, Sun L, Zollei L, Nimec D, Snyder B, Grant PE (2018). Reorganization of the somatosensory cortex in hemiplegic cerebral palsy associated with impaired sensory tracts. NeuroImage: Clinical.

[CR55] Papadopoulos N, McGinley J, Tonge B, Bradshaw J, Saunders K, Murphy A, Rinehart N (2012). Motor proficiency and emotional/behavioural disturbance in autism and Asperger’s disorder: another piece of the neurological puzzle?. Autism.

[CR56] Paquet A, Olliac B, Bouvard MP, Golse B, Vaivre-Douret L (2016). The semiology of motor disorders in autism spectrum disorders as highlighted from a standardized neuro-psychomotor assessment. Front Psychol.

[CR57] Paquet A, Golse B, Girard M, Olliac B, Vaivre-Douret L (2017). Laterality and lateralizationin autism spectrum disorder, using a standardized neuro-psychomotor assessment. Dev Neuropsychol.

[CR58] Paquet A, Olliac B, Golse B, Vaivre-Douret L (2019). Nature of motor impairments in autism spectrum disorder: a comparison with developmental coordination disorder. J Clin Exp Neuropsychol.

[CR59] Pitzianti M, D’Agati E, Casarelli L, Pontis M, Kaunzinger I, Lange KW, Tucha O, Curatolo P, Pasini A (2016). Neurological soft signs are associated with attentional dysfunction in children with attention deficit hyperactivity disorder. Cognit Neuropsychiatry.

[CR60] Plow EB, Varnerin N, Cunningham DA, Janini D, Bonnett C, Wyant A, Hou J, Siemionow V, Wang XF, Machado AG, Yue GH (2014). Age-related weakness of proximal muscle studied with motor cortical mapping: a TMS study. PLoS ONE.

[CR61] Redcay E, Courchesne E (2005). When is the brain enlarged in autism? A meta-analysis of all brain size reports. Biol Psychiatry.

[CR62] Rojas DC, Peterson E, Winterrowd E, Reite ML, Rogers SJ, Tregellas JR (2006). Regional gray matter volumetric changes in autism associated with social and repetitive behavior symptoms. BMC Psychiatry.

[CR63] Rossi S, Hallett M, Rossini PM, Pascual-Leone A, TMS Safety Consensus Group (2009). Safety, ethical considerations, and application guidelines for the use of transcranial magnetic stimulation in clinical practice and research. Clin Neurophys.

[CR64] Ruohonen J, Karhu J (2010). Navigated transcranial magnetic stimulation. Neurophysiol Clin.

[CR65] Sahlander C, Mattsson M, Bejerot S (2008). Motor function in adults with Asperger’s disorder: a comparative study. Physiother Theory Pract.

[CR66] Säisänen L, Pirinen E, Teitti S, Könönen M, Julkunen P, Määttä S, Karhu J (2008). Factors influencing cortical silent period: optimized stimulus location, intensity and muscle contraction. J Neurosci Methods.

[CR67] Säisänen L, Julkunen P, Niskanen E, Danner N, Hukkanen T, Lohioja T, Nurkkala J, Mervaala E, Karhu J, Könönen M (2008). Motor potentials evoked by navigated transcranial magnetic stimulation in healthy subjects. J Clin Neurophysiol.

[CR68] Säisänen L, Könönen M, Julkunen P, Määttä S, Vanninen R, Immonen A, Jutila L, Kälviäinen R, Jääskeläinen JE, Mervaala E (2010). Non-invasive preoperative localization of primary motor cortex in epilepsy surgery by navigated transcranial magnetic stimulation. Epilepsy Res.

[CR69] Säisänen L, Julkunen P, Lakka T, Lindi V, Könönen M, Määttä S (2018). Development of corticospinal motor excitability and cortical silent period from mid-childhood to adulthood—a navigated TMS study. Neurophysiol Clin.

[CR70] Schabrun SM, Jones E, Elgueta Cancino EL, Hodges PW (2014). Targeting chronic recurrent low back pain from the top-down and the bottom-up: a combined transcranial direct current stimulation and peripheral electrical stimulation intervention. Brain Stimul.

[CR71] Schwenkreis P, El Tom S, Ragert P, Pleger B, Tegenthoff M, Dinse HR (2007). Assessment of sensorimotor cortical representation asymmetries and motor skills in violin players. Eur J Neurosci.

[CR72] Shafer RL, Newell KM, Lewis MH, Bodfish JW (2017). A cohesive framework for motor stereotypy in typical and atypical development: the role of sensorimotor integration. Front Integr Neurosci.

[CR73] Shibasaki H (2012). Cortical activities associated with voluntary movements and involuntary movements. Clin Neurophys.

[CR74] Siebner HR, Bergman TO, Bestmann S, Massimini M, Johansen-Berg H, Mochizuki H, Bohning DE (2009). Consensus paper: combining transcranial stimulation with neuroimaging. Brain Stimul.

[CR75] Steenhuis RE, Bryden MP, Schwartz M, Lawson S (1990). Reliability of hand preference items and factors. J Clin Exp Neuropsychol.

[CR76] Strother L, Medendorp WP, Coros AM, Vilis T (2012). Double representation of the wrist and elbow in human motor cortex. Eur J Neurosci.

[CR77] Teitti S, Määttä S, Säisänen L, Könönen M, Vanninen R, Hannula H, Mervaala E, Karhu J (2008). Non-primary motor areas in the human frontal lobe are connected directly to hand muscles. Neuroimage.

[CR78] Theoret H, Halligan E, Kobayashi M, Fregni F, Tager-Flusberg H, Pascual-Leone A (2005). Impaired motor facilitation during action observation in individuals with autism spectrum disorder. Curr Biol.

[CR79] Thompson A, Murphy D, Dell’Acqua F, Ecker C, McAlonan G, Howells H, Baron-Cohen S, Lai MC, Lombardo MV (2017). Impaired communication between the motor and somatosensory homunculus is associated with poor manual dexterity in autism spectrum disorder. Biol Psychiatry.

[CR80] Triggs WJ, Subramanium B, Rossi F (1999). Hand preference and transcranial magnetic stimulation asymmetry of cortical motor representation. Brain Res.

[CR81] Tyc F, Boyadjian A, Devanne H (2005). Motor cortex plasticity induced by extensive training revealed by transcranial magnetic stimulation in human. Eur J Neurosci.

[CR82] Vaalto S, Julkunen P, Säisänen L, Könönen M, Määttä S, Karhu J (2013). Long-term plasticity may be manifested as reduction or expansion of cortical representations of actively used muscles in motor skill specialists. NeuroReport.

[CR83] Vitikainen AM, Salli E, Lioumis P, Mäkelä JP, Metsähonkala L (2013). Applicability of nTMS in locating the motor cortical representation areas in patients with epilepsy. Acta Neurochir.

[CR84] Wassermann EM, McShane LM, Hallett M, Cohen LG (1992). Noninvasive mapping of muscle representations in human motor cortex. Electroencephalogr Clin Neurophysiol.

[CR85] Wilke M, Holland SK, Altaye M, Gaser C (2008). Template-O-Matic: a toolbox for creating customized pediatric templates. NeuroImage.

