# Supplementary figures and images for: Functional and structural asymmetry in primary motor cortex in Asperger syndrome: a navigated TMS and imaging study
Source: Brain Topogr. 2019 Apr 4;32(3):504–18. doi: 10.1007/s10548-019-00704-0 (PMC6477009; doi:10.1007/s10548-019-00704-0)

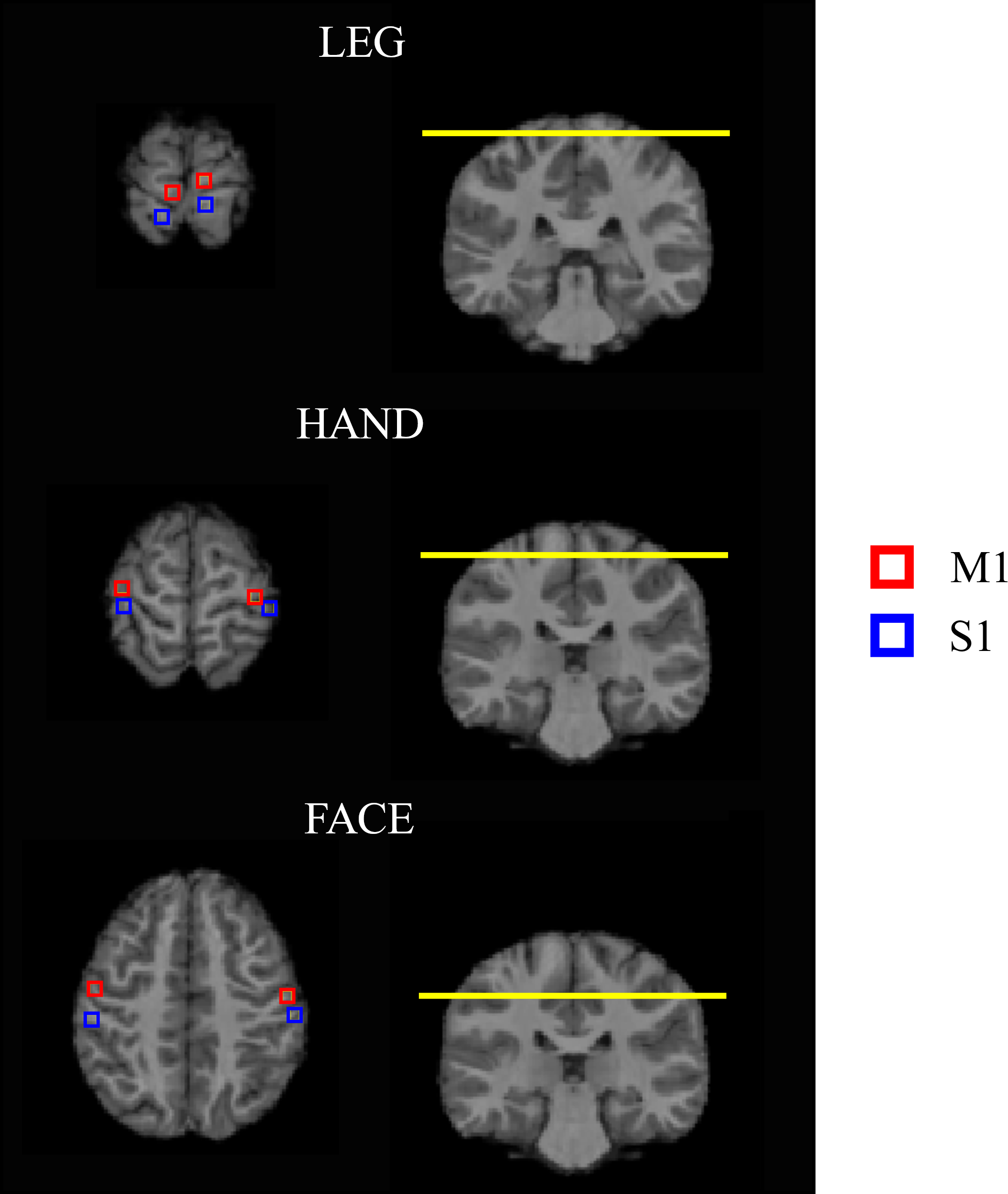

Supplement: Supplementary file 1 — Supplementary Figure—On the left, region-of interests (ROIs) of the leg, hand and face areas in both M1 (red) and primary sensory cortex (blue) from a representative subject. On the right, the level where the ROIs were placed in the coronal plane. Footnote: Of note. The ROIs cover several slices and the center of the ROI was not in the same slice in any of the subject. Thereby, as the aim of this figure was to visualize all ROIs in the same slice, the face ROI seems to be in WM, which is not the case. (TIF 1474 KB) [file 10548_2019_704_MOESM1_ESM.tif]
